# Autologous conditioned serum in treatment of periodontal diseases

**DOI:** 10.15171/japid.2019.008

**Published:** 2019-12-18

**Authors:** Adileh shirmohammadi

**Affiliations:** ^1^Department of Periodontics, Faculty of Dentistry, Tabriz University of Medical Sciences, Tabriz, Iran

Periodontal disease (PD) is an immuno-inflammatory condition, which is induced by the bacterial biofilm aggregates on external tooth surfaces. The condition affects and destroys the gingival tissue, periodontal ligament, cementum, and alveolar bone.^1^

Periodontopathogenic bacteria are the primary causative agents to destroy the supporting periodontal tissues directly through the action of their components, particularly the lipopolysaccharides (LPS) on the cell wall of gram-negative bacteria and in secreted molecules.^2^ Furthermore, in an indirect manner, the microorganisms can induce the host cells to express certain genes and release mediators, resulting in the exacerbation of the inflammatory response.^3^ The severity of PD is determined by the extent of tissue destruction caused by the immune response mounted by the host, probably influenced by environmental, acquired, or genetic risk factors.^4^

Treatment of PD should, therefore, involve the removal of dental biofilm and control of inflammation through scaling and root planing (SRP) as the conventional and essential procedures in periodontal therapy.^5^ Although SRP is the standard procedure for periodontal treatment, it might fail in eliminating bacteria or modulating the host response.

In recent years, a variety of new techniques and materials have been introduced to help regenerate periodontal tissues, and autologous conditioned serum** (**ACS) might be a promising material.

Periodontal therapy aims to regenerate periodontal tissues. However, current treatments, including surgery, use of membranes to facilitate periodontal tissue maturation, and application of enamel matrix derivatives, have limited indications and outcomes, prompting researchers to develop novel strategies for tissue engineering purposes. Several cytokines have been reported to play crucial roles in the destruction of periodontal tissues. However, their role in each phase of the periodontal wound healing process is yet to be clarified. A new horizon in tissue engineering processes of periodontal structures might involve efforts to control and modulate the inflammatory response of the host to affect the secretion of cytokines or activate/inhibit them in a time‐dependent and site-specific manner.^7^

ACS, as a new therapeutic agent, was introduced in the mid-1990s in the form of an injectable agent rich in endogenous IL-1Ra for osteoarthritis (OA). Meijer et al^6^ induced a dramatic and steep increase in the secretion of various anti-inflammatory cytokines (such as IL-1Ra) by exposing blood to glass beads. Such an observation laid the foundation for the production of ACS to be injected into intra-articular spaces in six sessions twice weekly for three weeks. This treatment modality is offered to human subjects in some European countries, and its application is even more widespread for equine OA since ACS significantly improves clinical lameness in horses, protecting cartilage against degradation.^8^

It seems the use of this material would be useful in procedures to regenerate periodontal tissues and treat periodontitis and peri-implantitis.
